# Supplementary material for: Human iPSC-based Modeling of Pulmonary Fibrosis Reveals p300/CBP Inhibition Suppresses Alveolar Transitional Cell State
Source: Nat Commun. 2026 Feb 12;17:1214. doi: 10.1038/s41467-026-68909-z (PMC12901050; doi:10.1038/s41467-026-68909-z)
Supplement: Supplementary file 1 — Supplementary Information [file 41467_2026_68909_MOESM1_ESM.pdf]

Supplementary Information

Human iPSC-based Modeling of Pulmonary Fibrosis Reveals p300/CBP Inhibition Suppresses Alveolar Transitional Cell State

Yusuke Tsutsui[1], Atsushi Masui[1], Satoshi Konishi[1], Taro Tsujimura[2], Mio Iwasaki[1], Takuya Yamamoto[1,2,3] & Shimpei Gotoh[1,*]

[1]Center for iPS Cell Research and Application (CiRA), Kyoto University, Kyoto 606-8507, Japan

[2]Institute for the Advanced Study of Human Biology (WPI-ASHBi), Kyoto University, Kyoto 606-8501, Japan

[3]Medical-risk Avoidance Based on iPS Cells Team, RIKEN Center for Advanced Intelligence Project (AIP), Kyoto 606-8507, Japan

*Correspondence: gotoh.shimpei.5m@cira.kyoto-u.ac.jp

Supplementary Figure

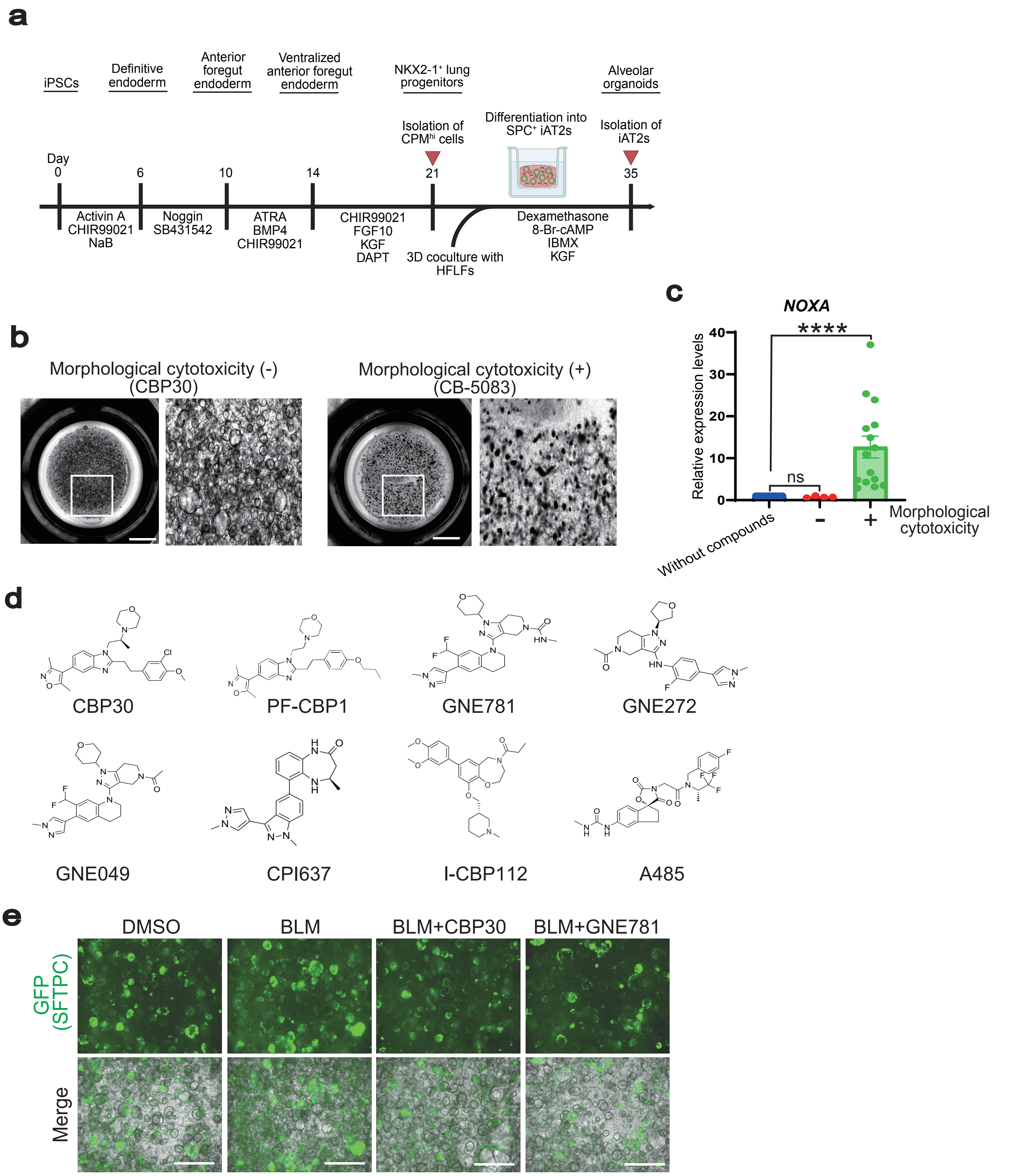

Supplementary Figure 1. Validation and characterization of the phenotypic screening for small molecule inhibitors of gel contraction in BLM-treated FD-AOs

a Schematic diagram of the stepwise differentiation from iPSCs into iAT2s. Created in BioRender. Tsutsui, Y. (2026) https://BioRender.com/c21opuf **b** Whole-well images of organoids exhibiting compound-induced cytotoxic morphological changes. Scale bars indicate 2 mm. **c** Gene expression data of the apoptosis marker *NOXA* in BLM FD-AOs. Data are presented as mean ± SEM. n = 9 (Without compound), n = 4 (Cytotoxicity(-)), and n = 15 (Cytotoxicity(+)) biologically independent experiments. One-way ANOVA followed by Tukey`s multiple comparisons test; ****p* < 0.001. ns, not significant. **d** Structures of p300/CBP inhibitors. **e** Live-cell imaging of FD-AOs. Scale bar, 1 mm.

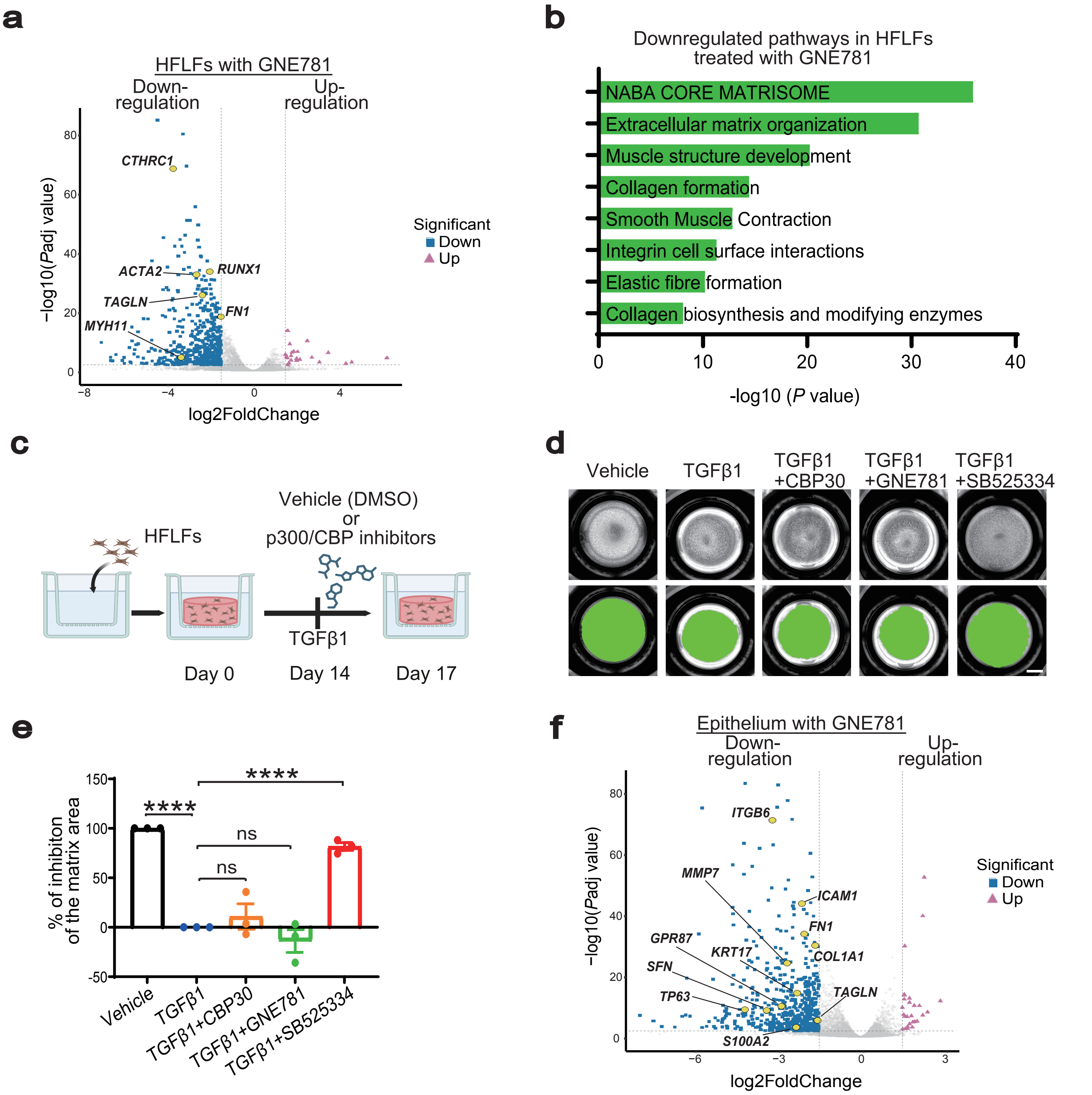

Supplementary Figure 2. GNE781, a p300/CBP inhibitor with a chemotype different from CBP30, also inhibits fibroblast activation and reduces ATCS induction in a BLM-induced pulmonary fibrosis model using FD-AOs.

a Volcano plot generated from the DESeq2 analysis of EpCAM$^-$ cells, with and without treatment from GNE781 (n = 3 biologically independent experiments). Thresholds: $|log_2FC| \geq 1.5$ and *p*adj ≤ 0.01 (dashed lines). EpCAM$^-$ cells were isolated from the BLM-induced pulmonary fibrosis model of FD-AOs. **b** Gene Ontology (GO) analysis utilizing the top 500 differentially expressed genes (DEGs) following BLM treatment in EpCAM$^-$ cells. **c** Schematic outline for the evaluation of p300/CBP inhibitors in the TGFβ 1-induced gel contraction of HFLFs-only 3D culture matrices.Created in BioRender. Tsutsui, Y. (2026) https://BioRender.com/c68d733 **d** Whole-well imaging of the 3D-cultured fibroblast area on Day 17. Each well contained 3 ng/mL of active TGFβ1 and was treated with either 10 μM p300/CBP inhibitors or 1 μM SB525334 (positive control) from days 14 to 17. Scale bars indicate 2 mm. **e** Quantification of the matrix area. Data are presented as mean ± SEM. Statistical analysis was performed using one-way ANOVA followed by Tukey's multiple comparisons test; ****$p < 0.0001$ (n = 3 biologically independent experiments). **f** Volcano plot generated from the DESeq2 analysis of EpCAM$^+$ cells, with and without treatment from GNE781 (n = 3 biologically independent experiments). Thresholds: $|log_2FC| \geq 1.5$ and *p*adj ≤ 0.01 (dashed lines). EpCAM$^+$ cells were isolated from the BLM-induced pulmonary fibrosis model of FD-AOs.

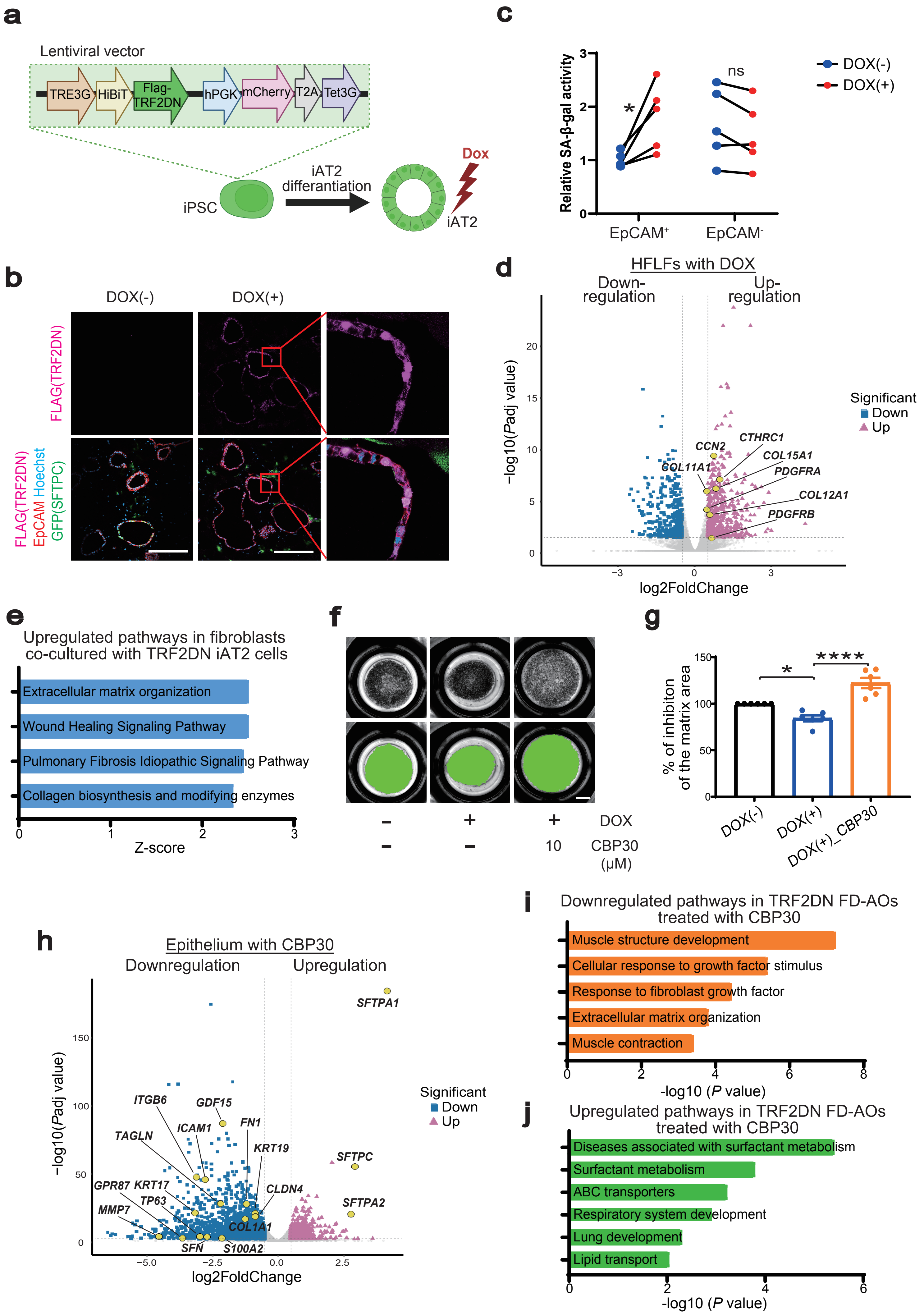
a
Lentiviral vector
TRE3G
HiBiT
Flag-TRF2DN
hPGK
mCherry
T2A
Tet3G
iPSC
iAT2 differantiation
Dox
iAT2
c
Relative SA-β-gal activity
*
ns
DOX(-)
DOX(+)
EpCAM+
EpCAM-
b
DOX(-)
DOX(+)
FLAG(TRF2DN)
FLAG(TRF2DN) EpCAM Hoechst GFP(SFTPC)
d
HFLFs with DOX
Down-regulation
Up-regulation
−log10(Padj value)
log2FoldChange
Significant
Down
Up
CCN2
CTHRC1
COL15A1
COL11A1
PDGFRA
COL12A1
PDGFRB
e
Upregulated pathways in fibroblasts co-cultured with TRF2DN iAT2 cells
Extracellular matrix organization
Wound Healing Signaling Pathway
Pulmonary Fibrosis Idiopathic Signaling Pathway
Collagen biosynthesis and modifying enzymes
Z-score
f
DOX
CBP30 (μM)
10
g
% of inhibiton of the matrix area
*
****
DOX(-)
DOX(+)
DOX(+)_CBP30
h
Epithelium with CBP30
Downregulation
Upregulation
−log10(Padj value)
log2FoldChange
Significant
Down
Up
SFTPA1
ITGB6
GDF15
FN1
ICAM1
TAGLN
KRT19
SFTPC
GPR87
KRT17
CLDN4
SFTPA2
TP63
MMP7
COL1A1
SFN
S100A2
i
Downregulated pathways in TRF2DN FD-AOs treated with CBP30
Muscle structure development
Cellular response to growth factor stimulus
Response to fibroblast growth factor
Extracellular matrix organization
Muscle contraction
-log10 (P value)
j
Upregulated pathways in TRF2DN FD-AOs treated with CBP30
Diseases associated with surfactant metabolism
Surfactant metabolism
ABC transporters
Respiratory system development
Lung development
Lipid transport
-log10 (P value)

Supplementary Figure 3. A p300/CBP inhibitor improves the pulmonary fibrotic phenotype in a TRF2DN-induced pulmonary fibrosis model using FD-AOs.

a Schematic representation of the strategy used to generate an iPSC line that expresses TRF2DN in response to doxycycline (DOX). Created in BioRender. Tsutsui, Y. (2026) https://BioRender.com/o27i084 **b** Representative immunofluorescence images showing SPC-GFP, EpCAM, FLAG (TRF2DN), and nuclei (Hoechst) in the TRF2DN-induced pulmonary fibrosis model with FD-AOs. Scale bars represent 200 μm. Representative images from three independent experiments with similar results are shown. **c** SA-β-gal activity in isolated $EpCAM^{+}$ and $EpCAM^{-}$ cells. Each value was normalized based on protein concentration and expressed relative to $EpCAM^{+}$ cells not treated with DOX. Data are presented as mean ± SEM (n = 5 biologically independent experiments). Statistical analysis was performed using one-way ANOVA followed by Tukey's multiple comparisons test; $*p < 0.05$; ns, not significant. **d** Volcano plot derived from the DESeq2 analysis of $EpCAM^{-}$ cells with or without DOX (n = 3 biologically independent experiments). Thresholds: $|\log_2 FC| \geq 0.5$ and $p\text{adj} \leq 0.05$ (dashed lines). **e** Pathway enrichment analysis using the Ingenuity Pathway Analysis (IPA) software for genes upregulated by TRF2DN. The threshold for upregulation was set to differentially expressed genes with $p < 0.05$. **f** Whole-well imaging of the matrices from FD-AOs in the TRF2DN-induced pulmonary fibrosis model on day 17. Each well was treated with DOX from days 9 to day 17 and 10 μM CBP30 from days 14 to day 17. Concentration units for the compound are in μM. Scale bars represent 2 mm. **g** Quantification of the matrix areas of FD-AOs under each condition. Statistical analysis was performed using one-way ANOVA followed by Tukey's multiple comparisons test; $**p < 0.01$. n = 6 biologically independent experiments per condition. **h** Volcano plot derived from the DESeq2 analysis of $EpCAM^{+}$ cells with or without CBP30 (n = 3 biologically independent experiments). Thresholds: $|\log_2 FC| \geq 0.5$ and $p\text{adj} \leq 0.05$ (dashed lines). $EpCAM^{+}$ cells were isolated from the TRF2DN-induced pulmonary fibrosis model of FD-AOs. **i** Pathway enrichment analysis using the Reactome software for proteins downregulated by CBP30 treatment. Proteomic analysis was conducted on all cultivation matrices, including the cells of FD-AOs. Differentially expressed proteins with a threshold of $p < 0.05$ were considered downregulated (n = 3 biologically independent experiments). **j** Pathway enrichment analysis using the Reactome software for proteins upregulated by CBP30 treatment. Proteomic analysis was performed on all the cultivation matrices, including the cells of FD-AOs. Differentially expressed proteins with a threshold of $p < 0.05$ were considered upregulated. (n = 3 biologically independent experiments)

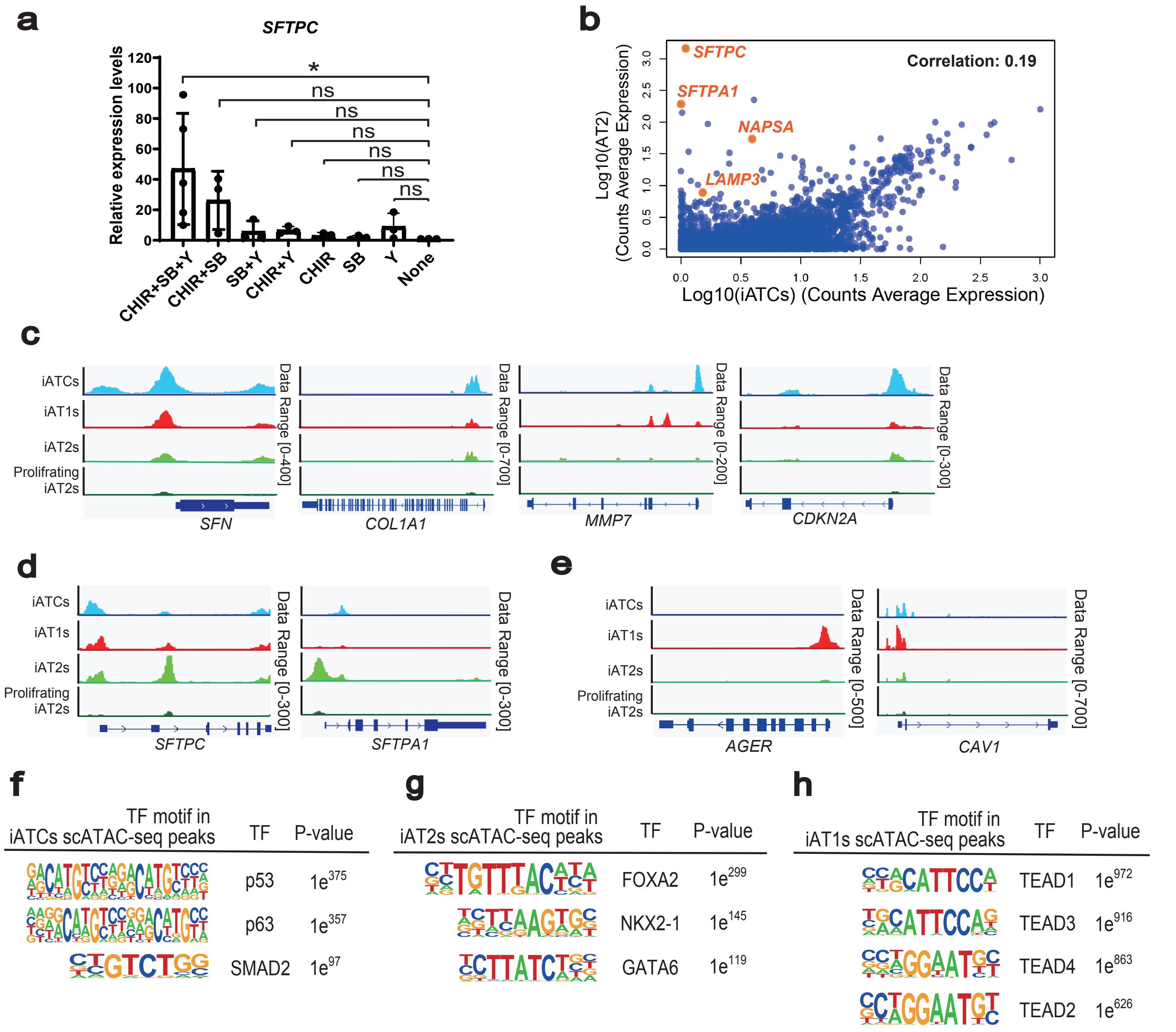

Supplementary Figure 4. Characterization of lineage marker expression and open chromatin accessibility in iATCs, iAT1s, and iAT2s.

a Gene expression of *SFTPC* in the micro-patterned culture is presented as mean ± SEM. n = 5 biologically independent experiments for CHIR+SB+Y, and n = 3 biologically independent experiments for all other conditions. Statistical analysis was performed using one-way ANOVA followed by Tukey's multiple comparisons test, with $*p < 0.05$. CHIR; CHIR99021, SB; SB431542, Y; Y27632. **b** Scatter plots comparing the gene expression profiles of AT2 cells from patients with pulmonary fibrosis (GSE135893)[1] and iATCs. The correlation coefficient was calculated using Pearson correlation. **c** Representative scATAC-seq tracks visualized in the Integrative Genomics Viewer (IGV) for the genes *SFN, COL1A1, MMP7,* and *CDKN2A* in each cluster of iATCs, iAT1s, and iAT2s. **d** Representative scATAC-seq tracks visualized in IGV for *SFTPC* and *SFTPA1* in each cluster of the iATCs, iAT1s, and iAT2s. **e** Representative scATAC-seq tracks visualized in IGV for *AGER* and *CAV1* in each cluster of the iATCs, iAT1s, and iAT2s. **f** Representative enriched transcription factor motifs identified in peaks that are significantly increased in the iATCs cluster compared to those of the iAT2s cluster from the scATAC-seq analysis. Differential accessibility was assessed using logistic regression–based analysis implemented in Seurat (FindMarkers, test.use = "LR"), with multiple-testing correction applied (adjusted *p*-value < 0.05). The same statistical analysis and significance criteria were applied in g and h. **g** Representative enriched transcription factor motifs identified in peaks that are significantly increased in the iAT2s cluster compared to the those of iAT1s cluster from the scATAC-seq analysis. **h** Representative enriched transcription factor motifs identified in peaks that are significantly increased in the iAT1s cluster compared to the those of iAT2s cluster from the scATAC-seq analysis.

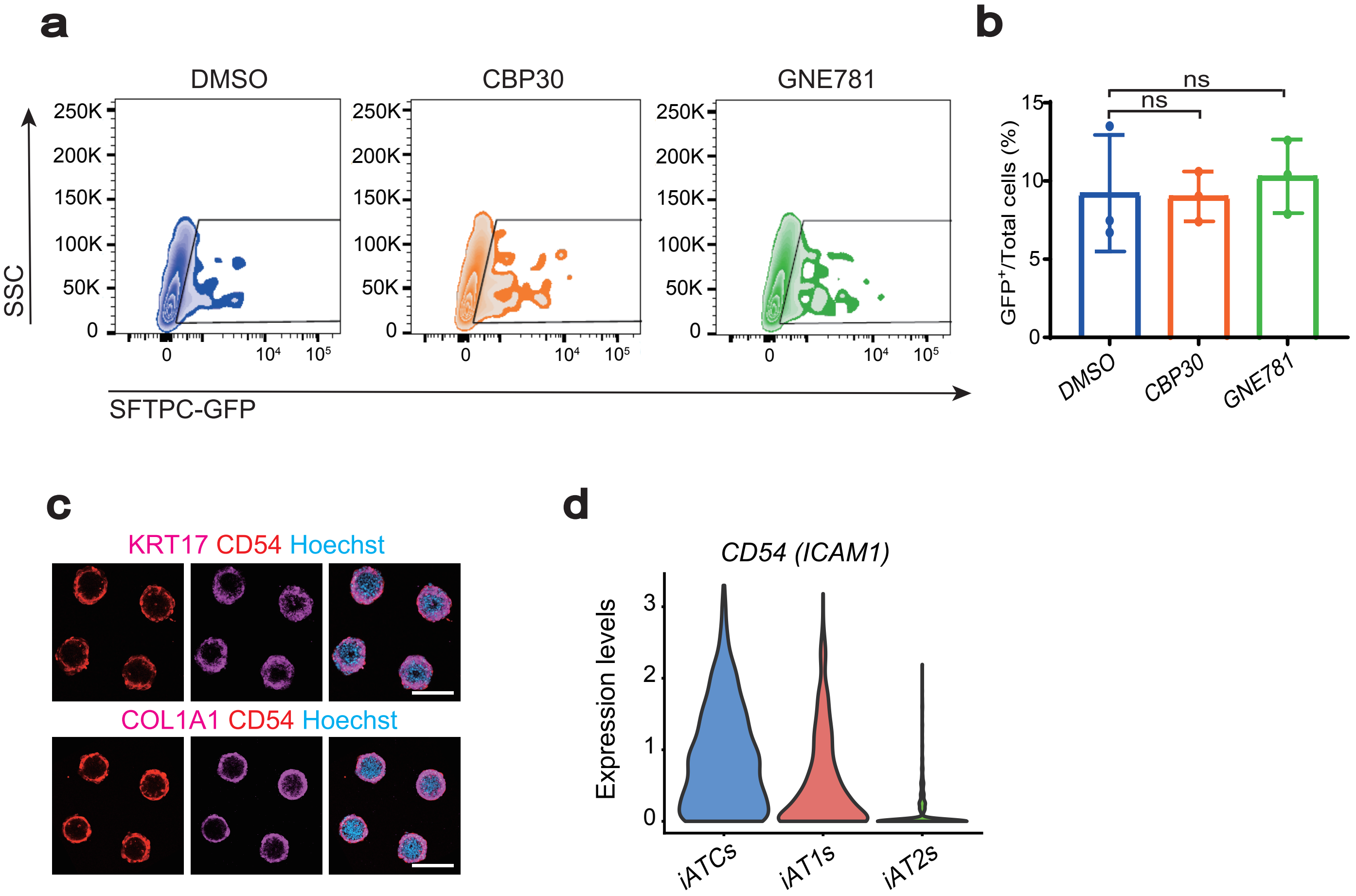

Supplementary Figure 5. Expression of CD54 in iATCs and evaluation of the effects of p300/CBP inhibitors on iAT2s differentiation.

a, b Flow cytometry analysis evaluated the SFTPC-GFP⁺ cell ratio in the micro-patterned culture. Each well was treated with p300/CBP inhibitors from days 11 to day 14. Data are presented as mean ± SEM. One-way ANOVA followed by Tukey's multiple comparisons test revealed no significant differences (ns) (n = 3 biologically independent experiments). **c** Immunostaining results for KRT17, COL1A1, CD54 (ICAM1), and nuclei (Hoechst) in the micro-patterned culture. Scale bar: 50 µm. Representative images from three independent experiments with similar results are shown. **d** Violin plots showing CD54 gene expression in iATCs, iAT1s, and iAT2s from the micro-patterned culture, corresponding to those shown in Fig. 4B.

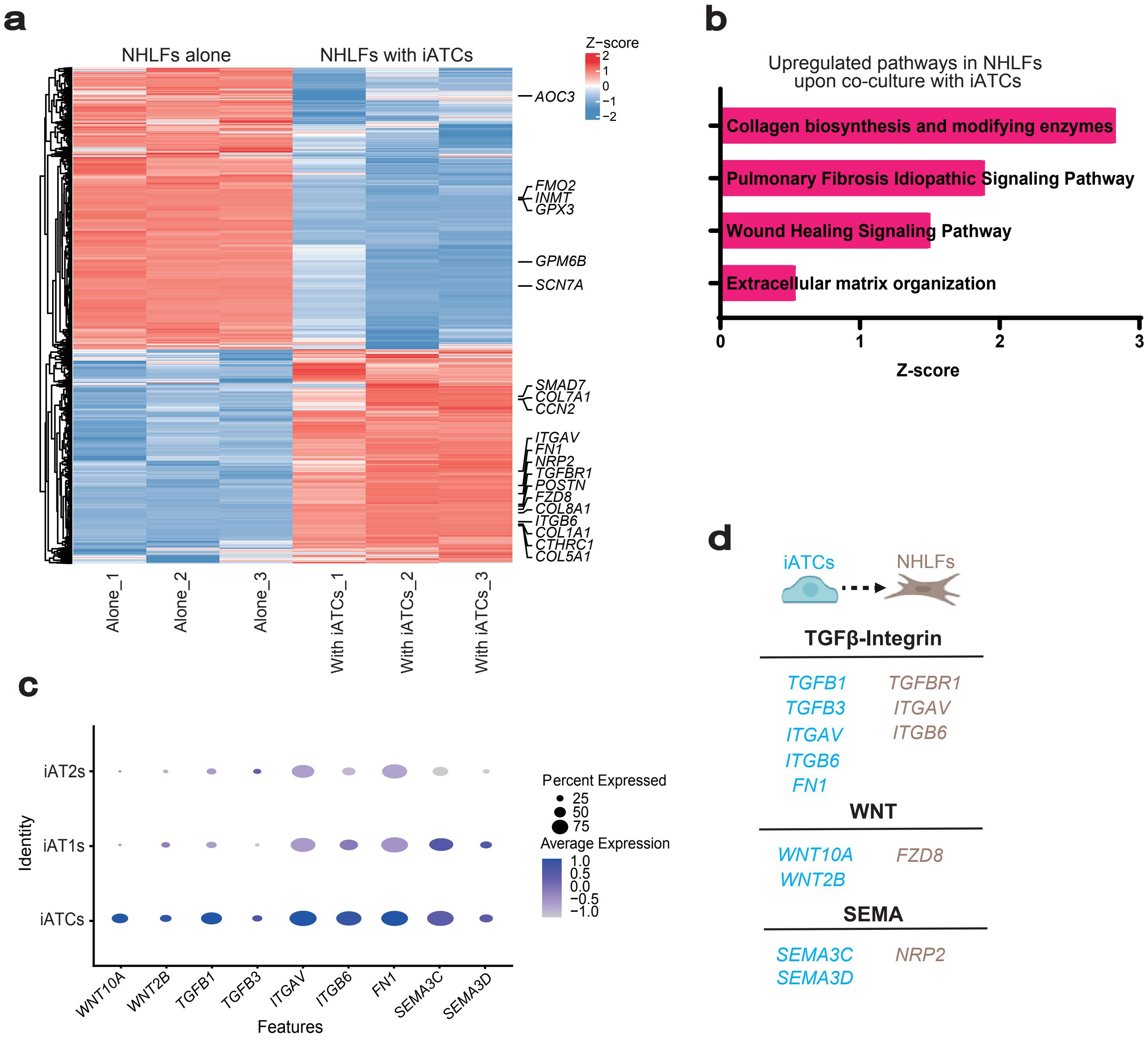

Supplementary Figure 6. Transcriptomic profiling of human adult lung fibroblasts co-cultured with iATCs. **a** Heatmap showing the top 2,000 differentially expressed genes identified by RNA-seq, ranked by the expression difference between NHLFs and NHLFs co-cultured with iATCs. **b** Pathway enrichment analysis using the IPA software for genes upregulated in lung fibroblasts co-cultured with iATCs. The threshold for upregulation was set to DEGs with $p < 0.05$. **c** Dot plots showing the expression of ligand candidates identified by human CellChat database[2] in each cluster. **d** Schematics of the receptor–ligand interactions between iATCs and NHLFs in the 2D co-culture model. Schematic illustrations of cell types (iATCs and NHLFs) were created using BioRender. All other elements were created by the authors. Created in BioRender. Tsutsui, Y. (2026) https://BioRender.com/yf1tyno

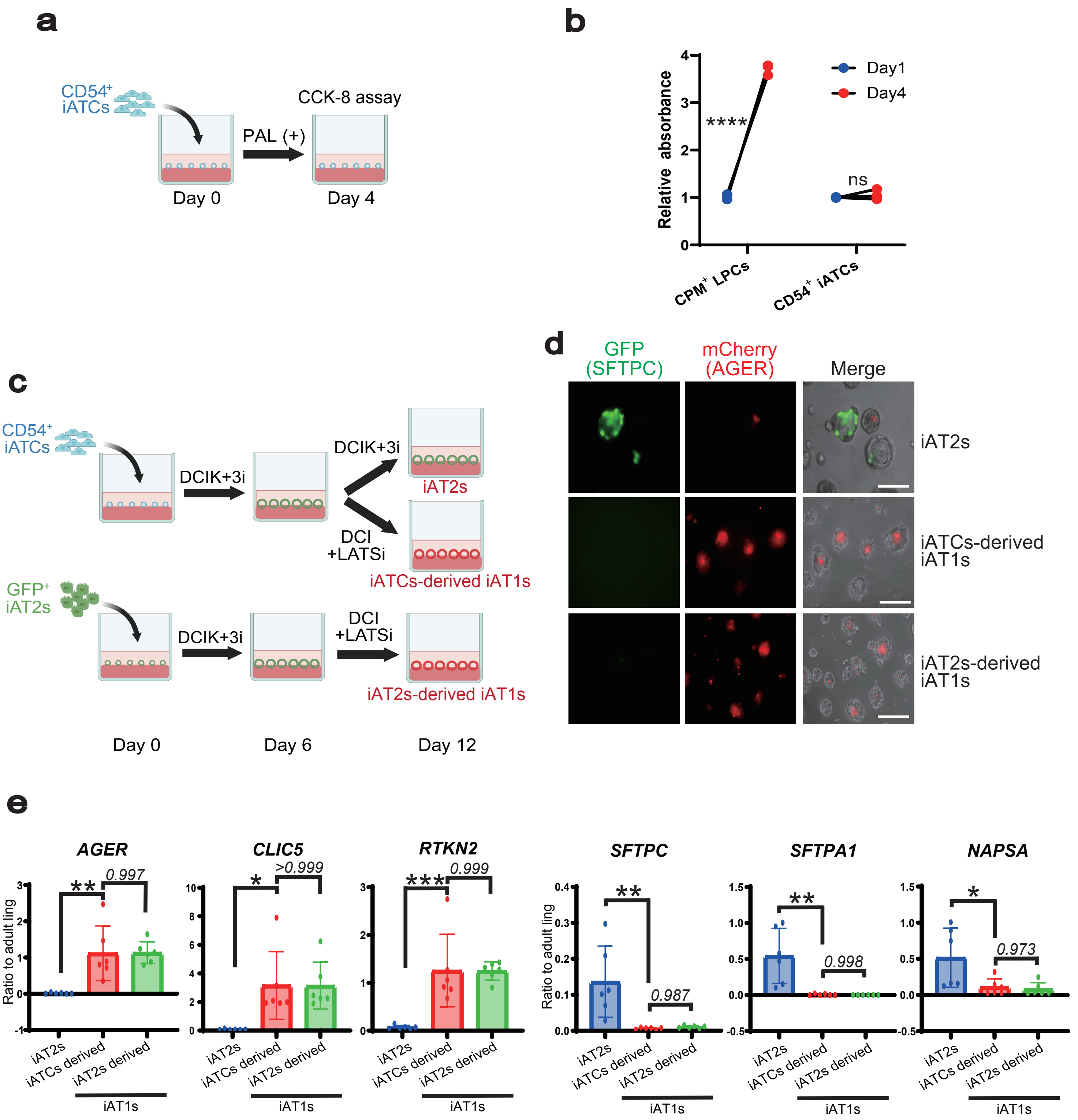

Supplementary Figure 7. iAT2s derived from iATCs possess the potential to differentiate into iAT1s.
a, b Evaluation of the self-renewal capacity of iATCs. (a) Experimental scheme. Created in BioRender. Tsutsui, Y. (2026) https://BioRender.com/hgv2msz (b) CCK-8 assay showing that iATCs do not proliferate. One-way ANOVA followed by Tukey's multiple comparisons test: ****$p < 0.0001$ (n = 3 biologically independent experiments). LPCs, lung progenitor cells. ns, not significant. **c** Schematic outline of the evaluation of differentiation potential from CD54⁺ iATCs to iAT2s or iAT1s, via iAT2s in on-gel culture. Created in BioRender. Tsutsui, Y. (2026) https://BioRender.com/xxf1erg. As a control, GFP⁺ iAT2s were differentiated into iAT1s. **d** Live-cell imaging of on-gel spheroids under each differentiation condition. Scale bars, 200 μm. Representative images from three independent experiments with similar results are shown. **e** Gene expression data of AT1 and AT2 markers in on-gel spheroids under each differentiation condition. Data are presented as mean ± SEM (n = 6 biologically independent experiments). One-way ANOVA followed by Tukey`s multiple comparisons test; *$p < 0.05$, **$p < 0.01$, ***$p < 0.001$. Numerical values indicate *p* values.

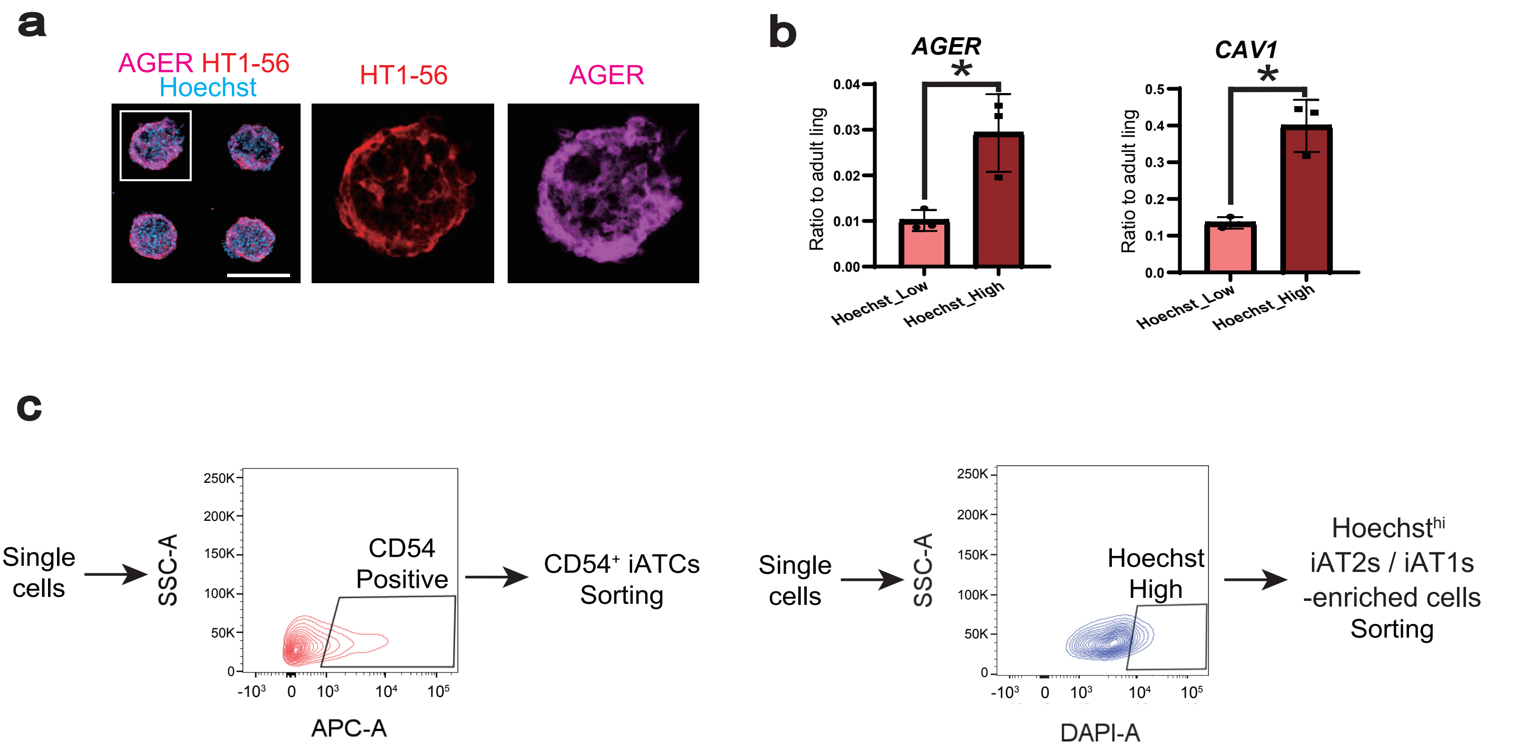

Supplementary Figure 8. Sorting strategy for iATCs, iAT2s, and iAT1s used for CUT&Tag.

a Immunostaining results for AGER, HT1-56, and nuclei (Hoechst) in the micro-patterned culture. Scale bar: 50 μm. Representative images from three independent experiments with similar results are shown. **b** Gene expression of AT1 markers in the micro-patterned culture. Data are presented as mean ± SEM (n = 3 biologically independent experiments). Unpaired two-tailed Student`s t test: *$p < 0.05$. **c** FACS gating strategy for $CD54^{+}$ iATCs (left) and $Hoechst^{hi}$ iAT2s / iAT1s-enriched cells (right).

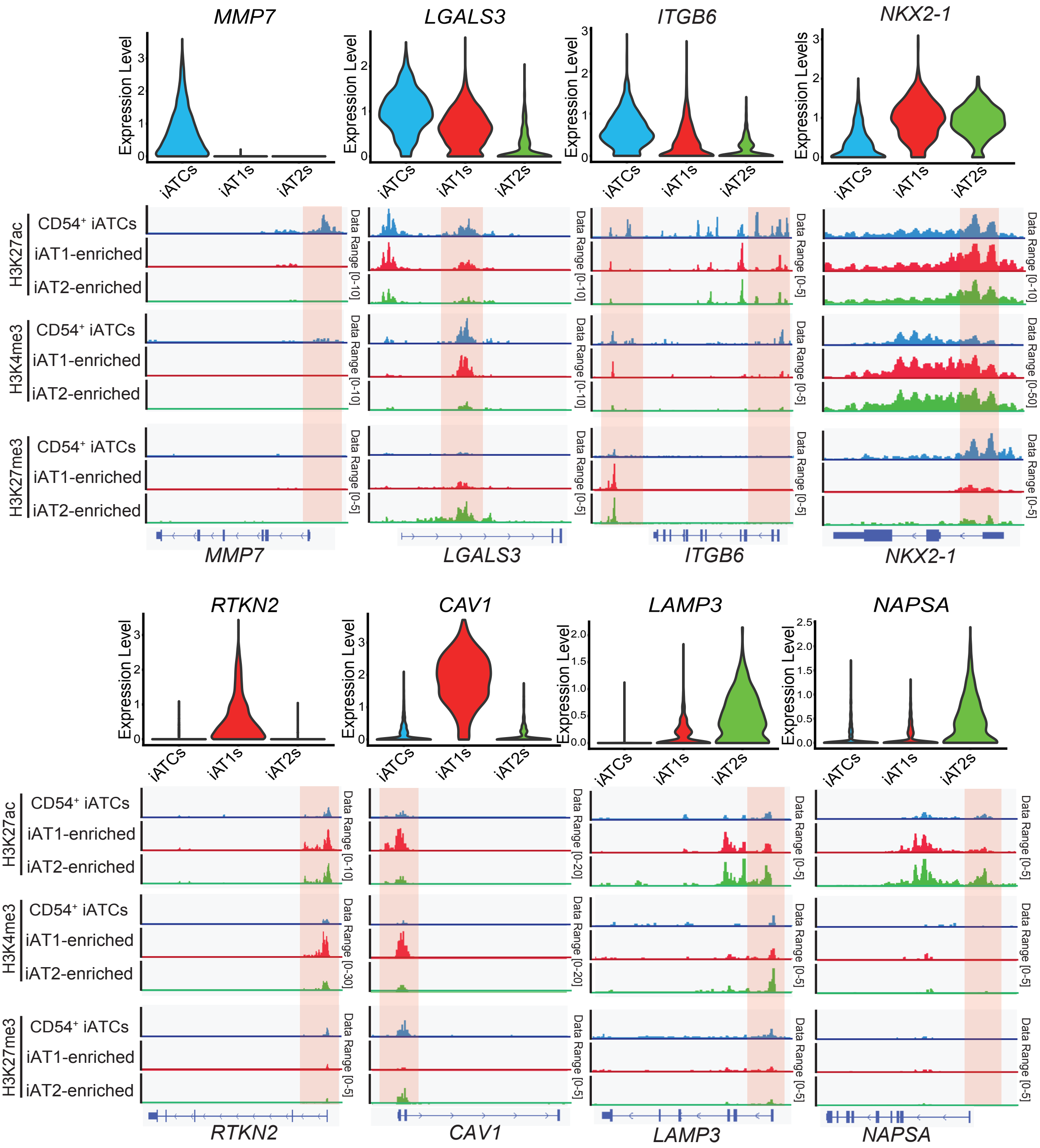

Supplementary Figure 9. Integrative Genomics Viewer (IGV) snapshots of lineage marker regions in iATCs, iAT1s, and iAT2s from CUT&Tag Analysis.
Violin plots of scRNA-seq expression from Figure 4b (top) and their corresponding CUT&Tag tracks in the IGV (bottom) for the genes *MMP7, LGALS3,* and *ITGB6* (iATCs markers), *NKX2-1* (lung epithelial marker), *RTKN2* and *CAV1* (iAT1s markers), and *LAMP3* and *NAPSA* (iAT2s markers).

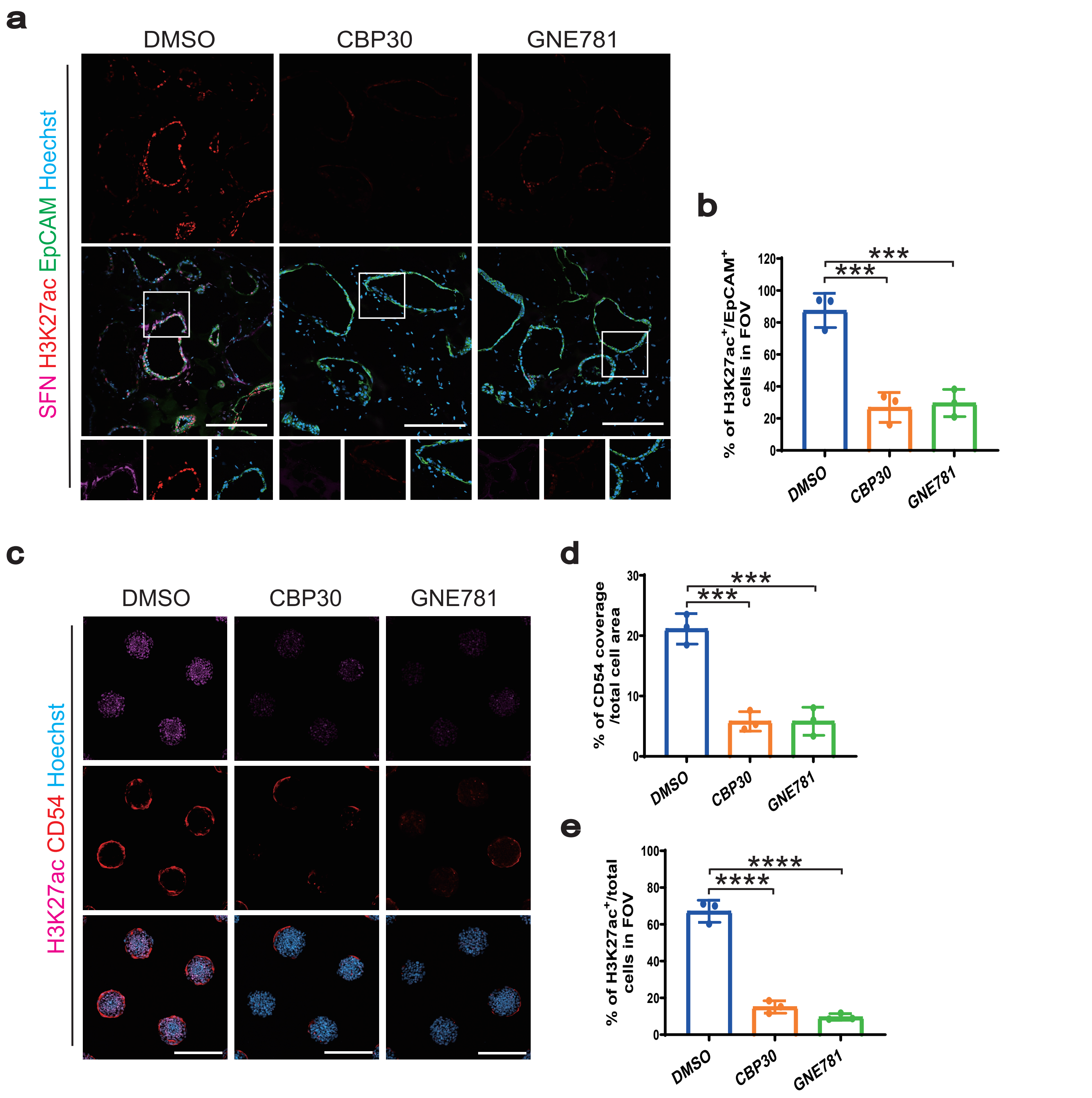

Supplementary Figure 10. H3K27ac suppressed by p300/CBP inhibitors in alveolar organoids.

a Immunostaining for H3K27ac and nuclei (Hoechst) was performed in the BLM-induced pulmonary fibrosis model of FD-AOs. Scale bar: 200 μm. **b** Quantification of the ratio of H3K27ac⁺ cells among EpCAM⁺ cells in the field of view (FOV) was conducted. One-way ANOVA followed by Tukey's multiple comparisons test showed significant results (****p < 0.001). (n = 3 biologically independent experiments). **c** Immunostaining for H3K27ac and nuclei (Hoechst) was also performed in the micro-patterned culture. Scale bar: 200 μm. **d, e** Quantification of the ratio of CD54 coverage and H3K27ac⁺ cells per total cell. One-way ANOVA followed by Tukey's multiple comparisons test revealed significant differences (***p < 0.001, ****p < 0.0001). (n = 3 biologically independent experiments).

a

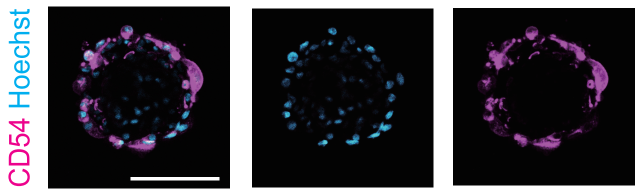

b

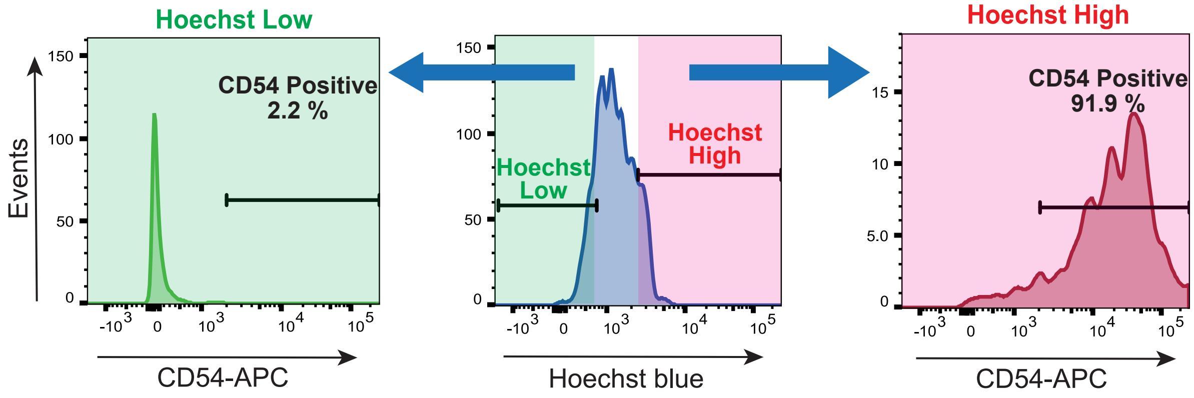

Supplementary Figure 11. Validation of CUT&Tag analysis using Hoechst-isolated iATCs treated with p300/CBP inhibitors. **a,b** Immunofluorescence (a) and flow cytometry analysis (b) to evaluate the enrichment of CD54⁺ cells by Hoechst staining. Scale bar: 100 µm.

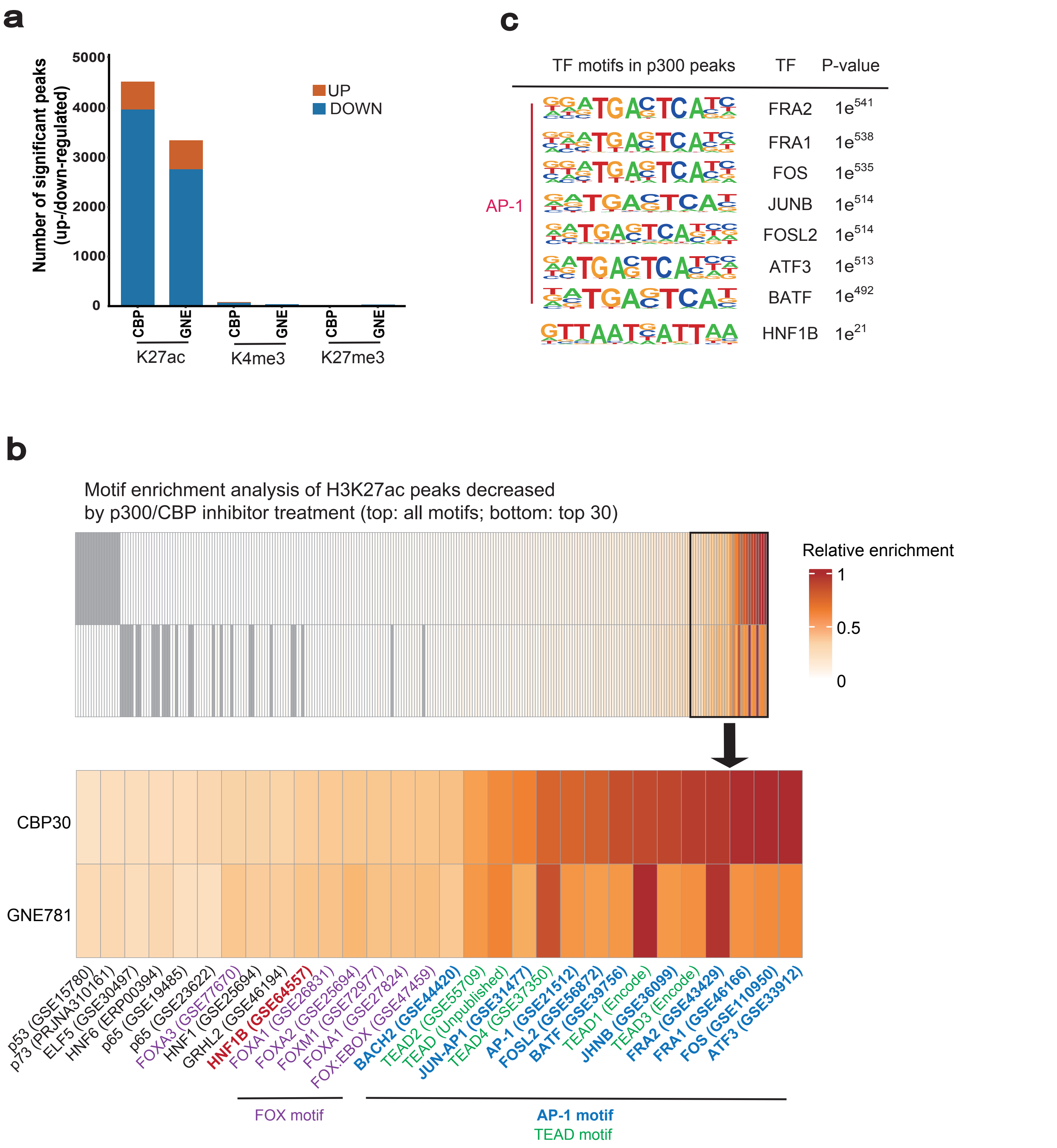

Supplementary Figure 12. Results of CUT&Tag analysis using Hoechst-isolated iATCs treated with p300/CBP inhibitors. **a** Number of significant peaks corresponding to Figure 8a. CBP; CBP30, GNE; GNE781. b Heatmap showing motif enrichment in H3K27ac peaks reduced by p300/CBP inhibitors (CBP30 and GNE781). The upper panel shows all detected motifs, and the lower panel displays the top 30 enriched motifs. The color scale indicates relative motif enrichment, calculated by dividing the $-\log_{10}(P)$ values by the maximum value within each inhibitor condition (0 = lowest, 1 = highest). Gray boxes indicate motifs not detected in that condition. **c** Representative enriched transcription factor motifs of p300 in iATCs-enriched cells.

a

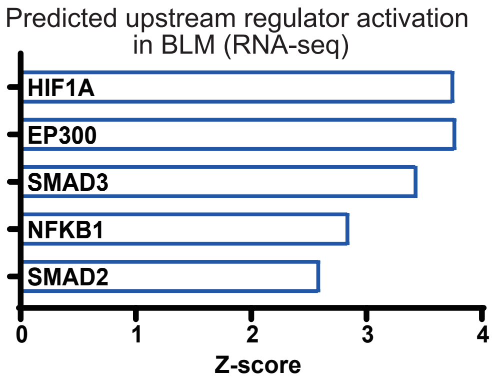

b

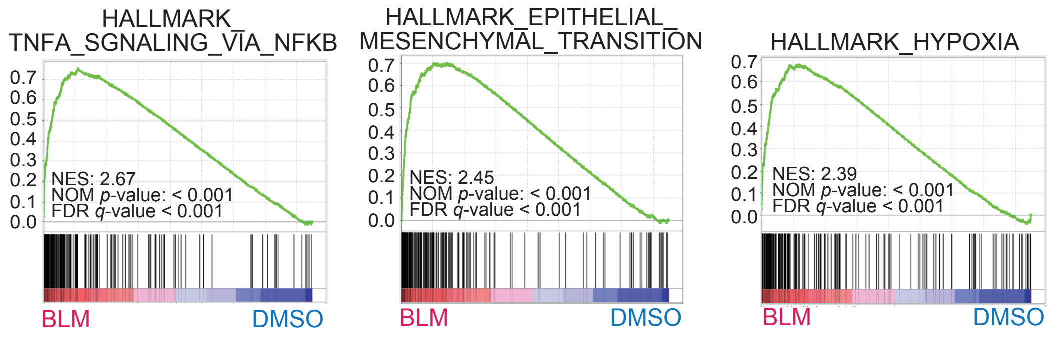

c

Transcription Factor Motif Enrichment in BLM-decreased Peaks

Day	Number of TF motif	TF
14	0	-
17	4	BRN1, TEAD3, EBF, NFE2L2

d

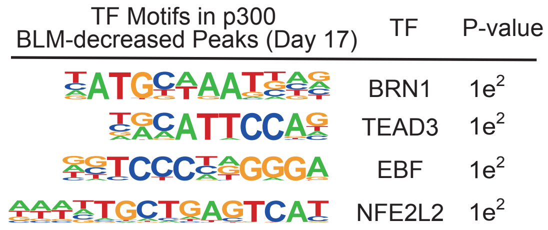

Supplementary Figure 13. Identification of upstream regulators and signaling pathways corresponding to p300 binding dynamics during ATCS differentiation.

a Upstream regulator analysis using the IPA software. The analysis was performed on DEGs upregulated by bleomycin treatment compared with DMSO control in the RNA-seq dataset shown in Figure 2. Predicted upstream regulators are ranked by their activation z-scores. **b** GSEA of DEGs upregulated by BLM treatment compared with DMSO control, using the Hallmark gene sets from the Molecular Signatures Database (MSigDB). Enriched pathways related to TNFα/NF-κB signaling, epithelial–mesenchymal transition, and hypoxia are shown. **c, d** Motif enrichment analysis of p300 peaks significantly decreased (adjusted *p* value < 0.05) upon BLM treatment in the epithelial cells isolated from FD-AOs. Significantly decreased peaks were identified using DiffBind. Motif enrichment was assessed using HOMER.

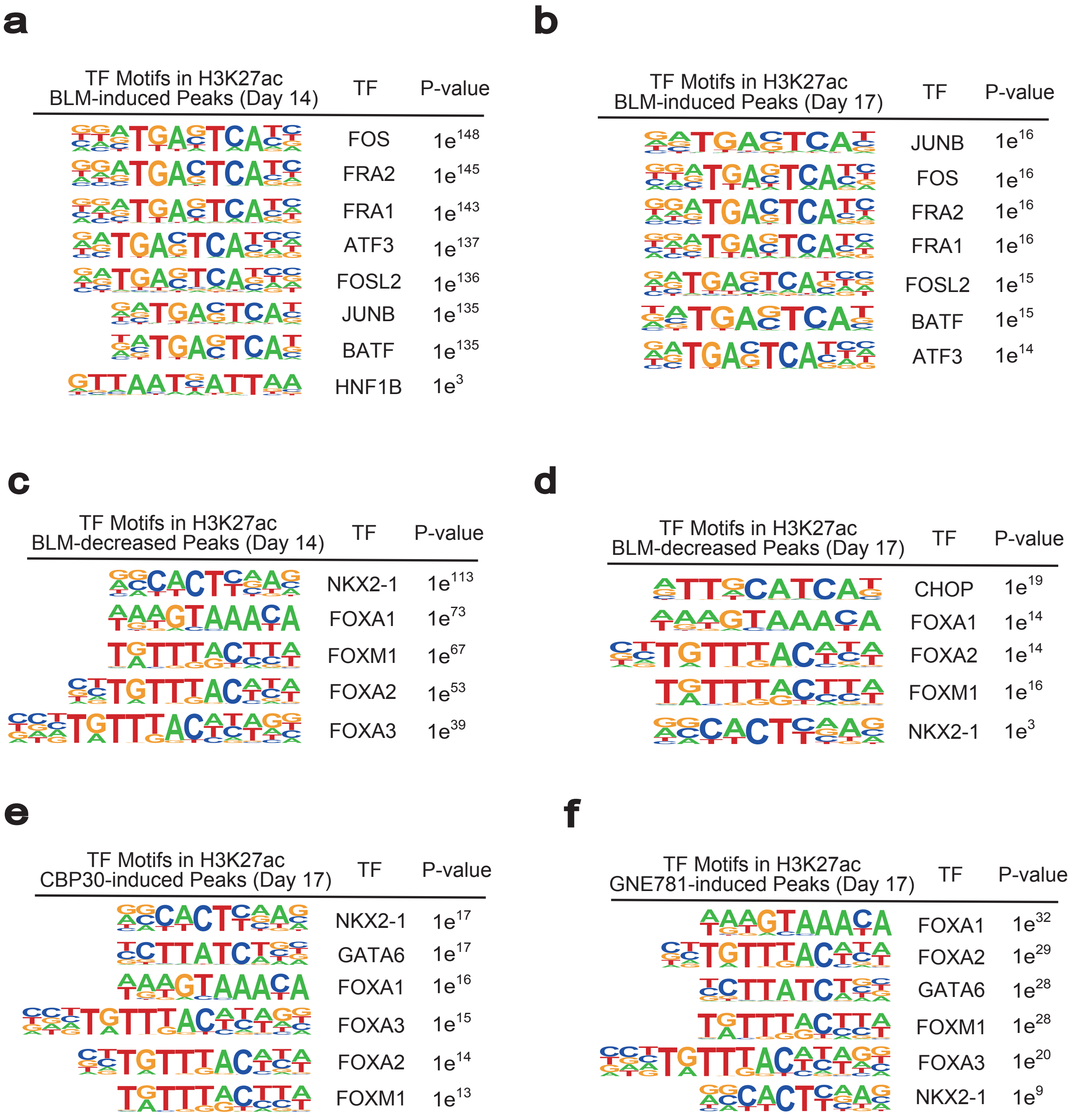

Supplementary Figure 14. Time course changes in motif enrichment of H3K27ac CUT&Tag peaks in bleomycin-treated FD-AOs.

a, b Representative enriched transcription factor motifs of H3K27ac peaks significantly increased (adjusted *p* value < 0.05) upon bleomycin treatment in the epithelial cells isolated from FD-AOs at day 14 (a) and day 17 (b). Significantly increased peaks were identified using DiffBind. Motif enrichment was assessed using HOMER. **c, d** Representative enriched transcription factor motifs of H3K27ac peaks significantly decreased (adjusted *p* value < 0.05) upon bleomycin treatment in the epithelial cells isolated from FD-AOs at day 14 (c) and day 17 (d). Significantly decreased peaks were identified using DiffBind. Motif enrichment was assessed using HOMER. **e, f** Representative enriched transcription factor motifs of H3K27ac peaks significantly increased (adjusted *p* value < 0.05) upon CBP30 (e) or GNE-781 (f) treatment in the epithelial cells isolated from FD-AOs. Significantly increased peaks were identified using DiffBind. Motif enrichment was assessed using HOMER.

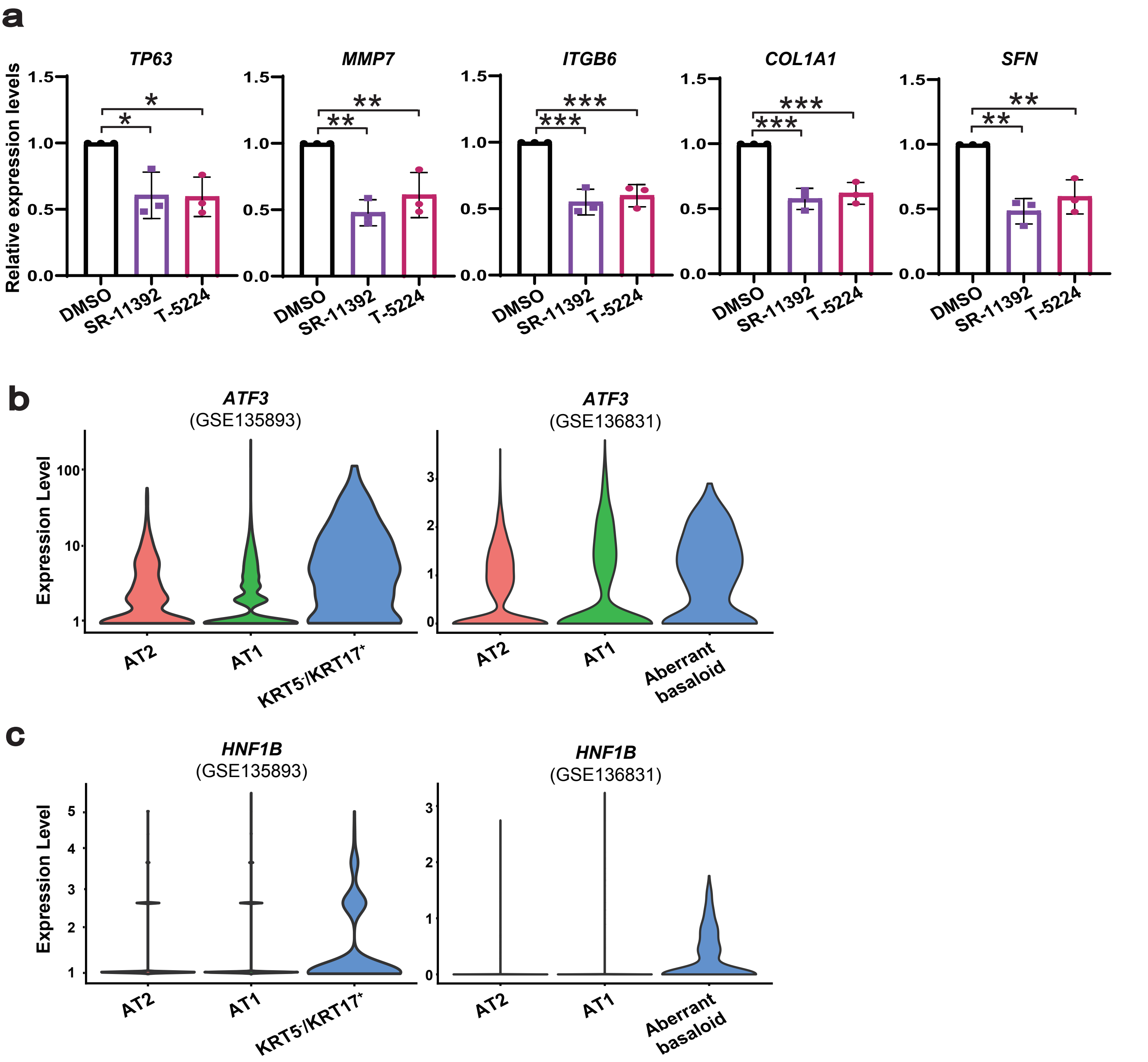

Supplementary Figure 15. AP-1 inhibition assay in iAT2-to-iATCs transition and expression of ATF3 and HNF1B in ATCS in human pulmonary fibrosis in the public database.

a Gene expression of ATCS markers in response to treatment of AP-1 inhibitors in the micro-patterned culture. Data are presented as mean ± SEM (n = 3 biologically independent experiments). One-way ANOVA followed by Tukey`s multiple comparisons test; ****p* < 0.001, *****p* < 0.0001. **b, c** Violin plots showing gene expression of *ATF3* (b) and *HNF1B* (c) in ATCS from previously published scRNA-seq datasets from patients with pulmonary fibrosis (GSE135893[1] and GSE136831[3]).

Supplementary Table

Supplementary Table 1. Gene lists used for lineage scoring in scRNA-seq analysis

ATCS	AT2	AT1
KRT17	*ABCA3*	*AGER*
MMP7	*ETV5*	*AQP5*
SOX4	*LAMP3*	*CAV1*
SFN	*NAPSA*	*PDPN*
ITGB6	*SFTPA1*	*CLIC5*
KRT8	*SFTPB*	*EMP2*
COL1A1	*SFTPC*	
CLDN4		
CDKN2A		
ICAM1		
FN1		
TP63		
LGAL3		

Supplementary Table 2. Oligonucleotide sequences used for qPCR in this study

Gene	Species	Forward	Reverse
Sfn	Mouse	AAGCGCATCATCGATTCTGC	TGGCTATCTCGTAGTGGAAGAC
Cldn4	Mouse	AGCCCTTATGGTCATCAGCATC	ATGCTTGCCACGATGAACAC
Krt19	Mouse	TTGCGCGACAAGATTCTTGG	CAGGCGAGCATTGTCAATCTG
Cthrc1	Mouse	AAGCAAAAAGCGCTGATCC	CCTGCTGGTCCTTGTAGACAC
Col1a1	Mouse	GCTCCTCTTAGGGGCCACT	CCACGTCTCACCATTGGGG
Col3a1	Mouse	TCACCAGGACAAAGAGGGGA	CCACCAGGACTGCCGTTATT
18s	Mouse	CTTTGGTCGCTCGCTCCTC	CTGACCGGGTTGGTTTTGAT
SOX4	Human	TTACCAGCTCCCTTCTTGCAG	AAAGCCTGCATGCAACAGAC
POSTN	Human	AACAGCAAACCACCTTCACG	ATTCACAGGTGCCAGCAAAG
HNF1B	Human	CACCTTGACGAATATCCACAGC	TGAGGCTTTGTGCAATTGCC
NAPSA	Human	AATATGGAACTGGGCGGGTAG	AAAATGGGCAAAAGCGAAGA
SLC34A2	Human	TCGCCACTGTCATCAAGAAG	CTCTGTACGATGAAGGTCATGC
CLIC5	Human	CTATGATATCCCGGCTGAGATGAC	CACGGGCATAGGCGTTCTT
CAV1	Human	AGGGCAACATCTACAAGCCC	GCCGTCAAAACTGTGTGTCC
RTKN2	Human	TTTTGAAGCCAAGCCAGTGC	TGCCTGCAGCCATGATCTAG
PMAIP1	Human	AACTCTTCTGCTCAGGAACCTG	CACCCATGAATGCACCTTCAC

Supplementary Table 3. TaqMan probes used for qPT-PCR in this study

Gene	TaqmanID
SFN	Hs00968567_s1
KRT17	Hs00356958_m1
MMP7	Hs01042796_m1
COL1A1	Hs00164004_m1
KRT19	Hs00761767_s1
ITGB6	Hs00168458_m1
TP63	Hs00978340_m1
CTHRC1	Hs00298917_m1
SFTPC	Hs00161628_m1
SFTPA1	Hs00831305_s1
AGER	Hs00542584_g1
ATF3	Hs00231069_m1
18s	Hs99999901_s1

References

1. Habermann, A. C. et al. Single-cell RNA sequencing reveals profibrotic roles of distinct epithelial and mesenchymal lineages in pulmonary fibrosis. *Sci Adv* **6**, eaba1972 (2020).
2. Jin, S. et al. Inference and analysis of cell-cell communication using CellChat. *Nat. Commun.* **12**, 1088 (2021).
3. Adams, T. S. et al. Single-cell RNA-seq reveals ectopic and aberrant lung-resident cell populations in idiopathic pulmonary fibrosis. *Sci Adv* **6**, eaba1983 (2020).
