# Supplementary material for: Human iPSC-based Modeling of Pulmonary Fibrosis Reveals p300/CBP Inhibition Suppresses Alveolar Transitional Cell State
Source: Nat Commun. 2026 Feb 12;17:1214. doi: 10.1038/s41467-026-68909-z (PMC12901050; doi:10.1038/s41467-026-68909-z)
Supplement: Supplementary file 2 — Description of Additional Supplementary Information [file 41467_2026_68909_MOESM2_ESM.pdf]

Description of Additional Supplementary Files

File Name: Supplementary Data 1
Description: List of compounds in the screening library.

File Name: Supplementary Data 2
Description: List of predicted target genes associated with H3K27ac inhibition in iATCs.

File Name: Supplementary Data 3
Description: Transcription Factor Motif Enrichment in BLM-Induced p300 Peaks in FDAOs.

File Name: Supplementary Data 4
Description: Oligonucleotide sequences used for CUT&Tag in this study.
