# Supplementary material for: Human iPSC-based Modeling of Pulmonary Fibrosis Reveals p300/CBP Inhibition Suppresses Alveolar Transitional Cell State
Source: Nat Commun. 2026 Feb 12;17:1214. doi: 10.1038/s41467-026-68909-z (PMC12901050; doi:10.1038/s41467-026-68909-z)
Supplement: Supplementary file 7 — Reporting Summary [file 41467_2026_68909_MOESM7_ESM.pdf]

nature portfolio

Corresponding author(s): Shimpei Gotoh

Last updated by author(s): Dec 26, 2025

Reporting Summary

Nature Portfolio wishes to improve the reproducibility of the work that we publish. This form provides structure for consistency and transparency in reporting. For further information on Nature Portfolio policies, see our Editorial Policies and the Editorial Policy Checklist.

Statistics

For all statistical analyses, confirm that the following items are present in the figure legend, table legend, main text, or Methods section.

n/a	Confirmed	
☐	☒	The exact sample size (*n*) for each experimental group/condition, given as a discrete number and unit of measurement
☐	☒	A statement on whether measurements were taken from distinct samples or whether the same sample was measured repeatedly
☐	☒	The statistical test(s) used AND whether they are one- or two-sided<br>*Only common tests should be described solely by name; describe more complex techniques in the Methods section.*
☒	☐	A description of all covariates tested
☐	☒	A description of any assumptions or corrections, such as tests of normality and adjustment for multiple comparisons
☐	☒	A full description of the statistical parameters including central tendency (e.g. means) or other basic estimates (e.g. regression coefficient) AND variation (e.g. standard deviation) or associated estimates of uncertainty (e.g. confidence intervals)
☐	☒	For null hypothesis testing, the test statistic (e.g. *F*, *t*, *r*) with confidence intervals, effect sizes, degrees of freedom and *P* value noted<br>*Give P values as exact values whenever suitable.*
☒	☐	For Bayesian analysis, information on the choice of priors and Markov chain Monte Carlo settings
☒	☐	For hierarchical and complex designs, identification of the appropriate level for tests and full reporting of outcomes
☐	☒	Estimates of effect sizes (e.g. Cohen's *d*, Pearson's *r*), indicating how they were calculated

Our web collection on statistics for biologists contains articles on many of the points above.

Software and code

Policy information about availability of computer code

Data collection	Images were taken by BZ-X710, BZ-X810 (Keyence), and LSM900 (Zeiss). qPCR analyses were performed using QuantStudio 3. FACS data were collected using FACS Aria III or FACS Symphony S6 (BD Biosciences).
Data analysis	Images were analyzed using Fiji v1.54 software.The gel area of FD-AOs was measured using cellSens software (Version 4, Olympus). Statistical analyses were performed using Prism10 (GraphPad). Flow cytometry data were analyzed using FlowJo v10.8.1. Single-cell RNA-seq data were processed using 10X Genomics Cell Ranger pipeline v7.1.0.Data were analyzed using Seurat v3.2.3 (R package). Single-cell ATAC-seq data were processed using Seurat and Signac (v1.13.0). Proteomic analysis data were analyzed with DIA-NN (v1.8.2 beta27).

For manuscripts utilizing custom algorithms or software that are central to the research but not yet described in published literature, software must be made available to editors and reviewers. We strongly encourage code deposition in a community repository (e.g. GitHub). See the Nature Portfolio guidelines for submitting code & software for further information.

Data

Policy information about availability of data

All manuscripts must include a data availability statement. This statement should provide the following information, where applicable:

- Accession codes, unique identifiers, or web links for publicly available datasets
- A description of any restrictions on data availability
- For clinical datasets or third party data, please ensure that the statement adheres to our policy

The accession numbers for bulk RNA-seq, bulk CUT & Tag, single-cell RNA-seq, and single-cell ATAC-seq data reported in this study are GSE289676 [https://www.ncbi.nlm.nih.gov/geo/query/acc.cgi?acc=GSE289676] , GSE289678 [https://www.ncbi.nlm.nih.gov/geo/query/acc.cgi?acc=GSE289678] , GSE289679 [https://www.ncbi.nlm.nih.gov/geo/query/acc.cgi?acc=GSE289679] , GSE289682 [https://www.ncbi.nlm.nih.gov/geo/query/acc.cgi?acc=GSE289682] , GSE289683 [https://www.ncbi.nlm.nih.gov/geo/query/acc.cgi?acc=GSE289683] , GSE291333 [https://www.ncbi.nlm.nih.gov/geo/query/acc.cgi?acc=GSE291333] , GSE289846 [https://www.ncbi.nlm.nih.gov/geo/query/acc.cgi?acc=GSE289846] , GSE290014 [https://www.ncbi.nlm.nih.gov/geo/query/acc.cgi?acc=GSE290014] , PRJDB37980 [https://ddbj.nig.ac.jp/search/entry/bioproject/ PRJDB37980] , PRJDB37982 [https://ddbj.nig.ac.jp/search/entry/bioproject/ PRJDB37982] , and PRJDB37983 [https://ddbj.nig.ac.jp/search/entry/bioproject/ PRJDB37983] , respectively. The proteomic analysis data was deposited to the ProteomeXchange Consortium via jPOSTrepo [https://repository.jpostdb.org/] with the dataset identifier JPST003570 (PXD060050).

Research involving human participants, their data, or biological material

Policy information about studies with human participants or human data. See also policy information about sex, gender (identity/presentation), and sexual orientation and race, ethnicity and racism.

Reporting on sex and gender	NA
Reporting on race, ethnicity, or other socially relevant groupings	NA
Population characteristics	NA
Recruitment	NA
Ethics oversight	NA

Note that full information on the approval of the study protocol must also be provided in the manuscript.

Field-specific reporting

Please select the one below that is the best fit for your research. If you are not sure, read the appropriate sections before making your selection.

☒ Life sciences ☐ Behavioural & social sciences ☐ Ecological, evolutionary & environmental sciences

For a reference copy of the document with all sections, see nature.com/documents/nr-reporting-summary-flat.pdf

Life sciences study design

All studies must disclose on these points even when the disclosure is negative.

Sample size	Sample size was decided based on the standard in the field.
Data exclusions	No data were excluded in this study.
Replication	The number of replicates is provided in the figure legends.
Randomization	For animal studies, mice were randomized based on body weight.
Blinding	Formal blinding was not implemented; however, all samples were treated and analyzed using the same standardized procedure.

Reporting for specific materials, systems and methods

We require information from authors about some types of materials, experimental systems and methods used in many studies. Here, indicate whether each material, system or method listed is relevant to your study. If you are not sure if a list item applies to your research, read the appropriate section before selecting a response.

Materials & experimental systems

n/a	Involved in the study	
☐	☒	Antibodies
☐	☒	Eukaryotic cell lines
☐	☒	Palaeontology and archaeology
☐	☒	Animals and other organisms
☒	☐	Clinical data
☒	☐	Dual use research of concern
☒	☐	Plants

Methods

n/a	Involved in the study	
☐	☒	ChIP-seq
☐	☒	Flow cytometry
☒	☐	MRI-based neuroimaging

Antibodies

Antibodies used

The antibodies used in this study were as follows:For fluorescence immunostaining, the following antibodies were used: GFP (Aves Labs, GFP-1020), SFN (Abcam, ab77187), act-p300 (biorbyt, ORB6262), EpCAM (Santa Cruz Biotechnology, sc-66020), KRT19 (Merck, MABT913), KRT17 (Abcam, ab109725), COL1A1 (Abcam, ab138492), CD54 (BioLegend, 353102), CD54 (Atlas, HPA002126), HT1-56 (Terrace Biotech, TB29AHT1-56), NaPi2b (kindly provided by Dr. Gerd Ritter (MX35)), AGER (R&D systems, AF1145), H3K27ac (Cell Signaling Technology, 8173), FLAG (Cell Signaling Technology, 14793), alpha smooth muscle Actin (Abcam, ab5694), and Fluorescin (Vector Laboratories, FL-1171). For flow cytometry, the following antibodies were used: Anti-CPM antibody (Fujifilm Wako, 014-27501), Anti-EPCAM-APC antibody (Miltenyi Biotec, 130-113-260), Anti-EpCAM antibody (Santa Cruz Biotechnology, SC-66020), and APC anti-CD54 antibody (BioLegend, 353112). For CUT&Tag, the following antibodies were used: Anti-H3K4me3 antibody (MAB Institute, MABI0304), Anti-H3K27me3 antibody (MAB Institute, MABI0323), Anti-H3 antibody (MAB Institute, MABI0301), p300 (Cell Signaling technology, #57625), and Anti-mouse IgG antibody (MAB Institute, ab46540).

Validation

Antibody validations are provided on manufacturers websites.

Eukaryotic cell lines

Policy information about cell lines and Sex and Gender in Research

Cell line source(s)

HFLFs (17.5 weeks of gestation; #PP002-F-1349, lot 121109VA) were purchased from DV Biologics.
NHLFs (37 years of age, Male, CC-2512, lot 20TL293907) were purchased from Lonza.
B2-3 was generated in our previous study (Gotoh S, et al., Stem Cell Rep, 2014).

Authentication

The identity of all cell lines was verified based on the provider's certification. B2-3 SPC-GFP hiPSC line was previously established (Gotoh S, et al. Stem Cell Rep, 2014) from its parental 201B7 iPSC line (Takahashi K, et al, Cell, 2007).

Mycoplasma contamination

Mycoplasma contamination was tested before use, and all cell lines were confirmed to be negative.

Commonly misidentified lines
(See ICLAC register)

None

Palaeontology and Archaeology

Specimen provenance

NA

Specimen deposition

NA

Dating methods

NA

☐ Tick this box to confirm that the raw and calibrated dates are available in the paper or in Supplementary Information.

Ethics oversight

NA

Note that full information on the approval of the study protocol must also be provided in the manuscript.

Animals and other research organisms

Policy information about studies involving animals; ARRIVE guidelines recommended for reporting animal research, and Sex and Gender in Research

Laboratory animals

Six-week-old male C57BL/6JJcl mice were obtained from CLEA Japan, Inc.

Wild animals

No wild animals were used in this study

Reporting on sex	Only male mice were used.
Field-collected samples	No field-collected samples were used.
Ethics oversight	All experiments involving animal use were performed per the guide for the care and use of laboratory animals of the Kyoto university, and the procedures were approved by the institutional committee of the Kyoto university (22-180-5).

Note that full information on the approval of the study protocol must also be provided in the manuscript.

Plants

Seed stocks	NA
Novel plant genotypes	NA
Authentication	NA

ChIP-seq

Data deposition

☒ Confirm that both raw and final processed data have been deposited in a public database such as GEO.

☒ Confirm that you have deposited or provided access to graph files (e.g. BED files) for the called peaks.

Data access links
May remain private before publication.

The accession numbers for bulk CUT & Tag reported in this study are GSE289683, GSE290014, PRJDB37980 and PRJDB37983. The tokens for the GSE datasets are “spevycyadrgfluh”, and “mjivykciblstfqt”.

Files in database submission

iATCs_H3K27ac_CUT1_R1.fastq.gz, iATCs_H3K27ac_CUT1_R2.fastq.gz, iAT2_H3K27ac_CUT1_R1.fastq.gz, iAT2_H3K27ac_CUT1_R2.fastq.gz, iAT1_H3K27ac_CUT1_R1.fastq.gz, iAT1_H3K27ac_CUT1_R2.fastq.gz, iATCs_H3K27ac_CUT2_R1.fastq.gz, iATCs_H3K27ac_CUT2_R2.fastq.gz, iAT2_H3K27ac_CUT2_R1.fastq.gz, iAT2_H3K27ac_CUT2_R2.fastq.gz, iAT1_H3K27ac_CUT2_R1.fastq.gz, iAT1_H3K27ac_CUT2_R2.fastq.gz, iATCs_H3K4me3_CUT1_R1.fastq.gz, iATCs_H3K4me3_CUT1_R2.fastq.gz, iAT1_H3K4me3_CUT1_R1.fastq.gz, iAT1_H3K4me3_CUT1_R2.fastq.gz, iAT2_H3K4me3_CUT1_R1.fastq.gz, iAT2_H3K4me3_CUT1_R2.fastq.gz, iATCs_H3K4me3_CUT2_R1.fastq.gz, iATCs_H3K4me3_CUT2_R2.fastq.gz, iAT1_H3K4me3_CUT2_R1.fastq.gz, iAT1_H3K4me3_CUT2_R2.fastq.gz, iAT2_H3K4me3_CUT2_R1.fastq.gz, iAT2_H3K4me3_CUT2_R2.fastq.gz, iATCs_H3K27me3_CUT1_R1.fastq.gz, iATCs_H3K27me3_CUT1_R2.fastq.gz, iAT1_H3K27me3_CUT1_R1.fastq.gz, iAT1_H3K27me3_CUT1_R2.fastq.gz, iAT2_H3K27me3_CUT1_R1.fastq.gz, iAT2_H3K27me3_CUT1_R2.fastq.gz, iATCs_H3K27me3_CUT2_R1.fastq.gz, iATCs_H3K27me3_CUT2_R2.fastq.gz, iAT1_H3K27me3_CUT2_R1.fastq.gz, iAT1_H3K27me3_CUT2_R2.fastq.gz, iAT2_H3K27me3_CUT2_R1.fastq.gz, iAT2_H3K27me3_CUT2_R2.fastq.gz, iATCs_H3_CUT1_R1.fastq.gz, iATCs_H3_CUT1_R2.fastq.gz, iAT2_H3_CUT1_R1.fastq.gz, iAT2_H3_CUT1_R2.fastq.gz, iAT1_H3_CUT1_R1.fastq.gz, iAT1_H3_CUT1_R2.fastq.gz, iATCs_H3_CUT2_R1.fastq.gz, iATCs_H3_CUT2_R2.fastq.gz, iAT2_H3_CUT2_R1.fastq.gz, iAT2_H3_CUT2_R2.fastq.gz, iAT1_H3_CUT2_R1.fastq.gz, iAT1_H3_CUT2_R2.fastq.gz, iATCs_H3K27ac_CUT1.bw, iAT2_H3K27ac_CUT1.bw, iAT1_H3K27ac_CUT1.bw, iATCs_H3K27ac_CUT2.bw, iAT2_H3K27ac_CUT2.bw, iAT1_H3K27ac_CUT2.bw, iATCs_H3K4me3_CUT1.bw, iATCs_H3K4me3_CUT2.bw, iAT1_H3K4me3_CUT1.bw, iAT1_H3K4me3_CUT2.bw, iAT2_H3K4me3_CUT1.bw, iAT2_H3K4me3_CUT2.bw, iATCs_H3K27me3_CUT1.bw, iATCs_H3K27me3_CUT2.bw, iAT1_H3K27me3_CUT1.bw, iAT1_H3K27me3_CUT2.bw, iAT2_H3K27me3_CUT1.bw, iAT2_H3K27me3_CUT2.bw, iATCs_H3_CUT1.bw, iAT2_H3_CUT1.bw, iAT1_H3_CUT1.bw, iATCs_H3_CUT2.bw, iAT2_H3_CUT2.bw, and iAT1_H3_CUT2.bw, iATCs_DMSO_CUT_1_H3_R1.fastq.gz, iATCs_DMSO_CUT_1_H3_R2.fastq.gz, ATCs_DMSO_CUT_1_H3K4me3_R1.fastq.gz, iATCs_DMSO_CUT_1_H3K4me3_R2.fastq.gz, iATCs_DMSO_CUT_1_H3K27me3_R1.fastq.gz, iATCs_DMSO_CUT_1_H3K27me3_R2.fastq.gz, iATCs_DMSO_CUT_1_H3K27ac_R1.fastq.gz, iATCs_DMSO_CUT_1_H3K27ac_R2.fastq.gz, iATCs_DMSO_CUT_1_p300_R1.fastq.gz, iATCs_DMSO_CUT_1_p300_R2.fastq.gz, iATCs_CBP30_CUT_1_H3_R1.fastq.gz iATCs_CBP30_CUT_1_H3_R2.fastq.gz, iATCs_CBP30_CUT_1_H3K4me3_R1.fastq.gz, iATCs_CBP30_CUT_1_H3K4me3_R2.fastq.gz, iATCs_CBP30_CUT_1_H3K27me3_R1.fastq.gz, iATCs_CBP30_CUT_1_H3K27me3_R2.fastq.gz, iATCs_CBP30_CUT_1_H3K27ac_R1.fastq.gz, iATCs_CBP30_CUT_1_H3K27ac_R2.fastq.gz, iATCs_GNE781_CUT_1_H3_R1.fastq.gz, iATCs_GNE781_CUT_1_H3_R2.fastq.gz, iATCs_GNE781_CUT_1_H3K4me3_R1.fastq.gz, iATCs_GNE781_CUT_1_H3K4me3_R2.fastq.gz, iATCs_GNE781_CUT_1_H3K27me3_R1.fastq.gz, iATCs_GNE781_CUT_1_H3K27me3_R2.fastq.gz, iATCs_GNE781_CUT_1_H3K27ac_R1.fastq.gz, iATCs_GNE781_CUT_1_H3K27ac_R2.fastq.gz, iATCs_DMSO_CUT_2_H3_R1.fastq.gz, iATCs_DMSO_CUT_2_H3_R2.fastq.gz, iATCs_DMSO_CUT_2_H3K4me3_R1.fastq.gz, iATCs_DMSO_CUT_2_H3K4me3_R2.fastq.gz, iATCs_DMSO_CUT_2_H3K27me3_R1.fastq.gz, iATCs_DMSO_CUT_2_H3K27me3_R2.fastq.gz, iATCs_DMSO_CUT_2_H3K27ac_R1.fastq.gz, iATCs_DMSO_CUT_2_H3K27ac_R2.fastq.gz, iATCs_DMSO_CUT_2_p300_R1.fastq.gz, iATCs_DMSO_CUT_2_p300_R2.fastq.gz, iATCs_CBP30_CUT_2_H3_R1.fastq.gz, iATCs_CBP30_CUT_2_H3_R2.fastq.gz, iATCs_CBP30_CUT_2_H3K4me3_R1.fastq.gz, iATCs_CBP30_CUT_2_H3K4me3_R2.fastq.gz, iATCs_CBP30_CUT_2_H3K27me3_R1.fastq.gz,

iATCs_CBP30_CUT_2_H3K27me3_R2.fastq.gz, iATCs_CBP30_CUT_2_H3K27ac_R1.fastq.gz, iATCs_CBP30_CUT_2_H3K27ac_R2.fastq.gz, iATCs_GNE781_CUT_2_H3_R1.fastq.gz, iATCs_GNE781_CUT_2_H3_R2.fastq.gz, iATCs_GNE781_CUT_2_H3K4me3_R1.fastq.gz, iATCs_GNE781_CUT_2_H3K4me3_R2.fastq.gz, iATCs_GNE781_CUT_2_H3K27me3_R1.fastq.gz, iATCs_GNE781_CUT_2_H3K27me3_R2.fastq.gz, iATCs_GNE781_CUT_2_H3K27ac_R1.fastq.gz, iATCs_GNE781_CUT_2_H3K27ac_R2.fastq.gz, Epithelial_DMSO_Day14_1_H3_R1.fastq.gz, Epithelial_DMSO_Day14_1_H3_R2.fastq.gz, Epithelial_DMSO_Day14_1_H3K27ac_R1.fastq.gz, Epithelial_DMSO_Day14_1_H3K27ac_R2.fastq.gz, Epithelial_DMSO_Day14_1_p300_R1.fastq.gz, Epithelial_DMSO_Day14_1_p300_R2.fastq.gz, Epithelial_BLM_Day14_1_H3_R1.fastq.gz, Epithelial_BLM_Day14_1_H3_R2.fastq.gz, Epithelial_BLM_Day14_1_H3K27ac_R1.fastq.gz, Epithelial_BLM_Day14_1_H3K27ac_R2.fastq.gz, Epithelial_BLM_Day14_1_p300_R1.fastq.gz, Epithelial_BLM_Day14_1_p300_R2.fastq.gz, Epithelial_DMSO_Day17_1_H3_R1.fastq.gz, Epithelial_DMSO_Day17_1_H3_R2.fastq.gz, Epithelial_DMSO_Day17_1_H3K27ac_R1.fastq.gz, Epithelial_DMSO_Day17_1_H3K27ac_R2.fastq.gz, Epithelial_DMSO_Day17_1_p300_R1.fastq.gz, Epithelial_DMSO_Day17_1_p300_R2.fastq.gz, Epithelial_BLM_Day17_1_H3_R1.fastq.gz, Epithelial_BLM_Day17_1_H3_R2.fastq.gz, Epithelial_BLM_Day17_1_H3K27ac_R1.fastq.gz, Epithelial_BLM_Day17_1_H3K27ac_R2.fastq.gz, Epithelial_BLM_Day17_1_p300_R1.fastq.gz, Epithelial_BLM_Day17_1_p300_R2.fastq.gz, Epithelial_CBP30_Day17_1_H3_R1.fastq.gz, Epithelial_CBP30_Day17_1_H3_R2.fastq.gz, Epithelial_CBP30_Day17_1_H3K27ac_R1.fastq.gz, Epithelial_CBP30_Day17_1_H3K27ac_R2.fastq.gz, Epithelial_GNE781_Day17_1_H3_R1.fastq.gz, Epithelial_GNE781_Day17_1_H3_R2.fastq.gz, Epithelial_GNE781_Day17_1_H3K27ac_R1.fastq.gz, Epithelial_GNE781_Day17_1_H3K27ac_R2.fastq.gz, Epithelial_DMSO_Day14_2_H3_R1.fastq.gz, Epithelial_DMSO_Day14_2_H3_R2.fastq.gz, Epithelial_DMSO_Day14_2_H3K27ac_R1.fastq.gz, Epithelial_DMSO_Day14_2_H3K27ac_R2.fastq.gz, Epithelial_DMSO_Day14_2_p300_R1.fastq.gz, Epithelial_DMSO_Day14_2_p300_R2.fastq.gz, Epithelial_BLM_Day14_2_H3_R1.fastq.gz, Epithelial_BLM_Day14_2_H3_R2.fastq.gz, Epithelial_BLM_Day14_2_H3K27ac_R1.fastq.gz, Epithelial_BLM_Day14_2_H3K27ac_R2.fastq.gz, Epithelial_BLM_Day14_2_p300_R1.fastq.gz, Epithelial_BLM_Day14_2_p300_R2.fastq.gz, Epithelial_DMSO_Day17_2_H3_R1.fastq.gz, Epithelial_DMSO_Day17_2_H3_R2.fastq.gz, Epithelial_DMSO_Day17_2_H3K27ac_R1.fastq.gz, Epithelial_DMSO_Day17_2_H3K27ac_R2.fastq.gz, Epithelial_DMSO_Day17_2_p300_R1.fastq.gz, Epithelial_DMSO_Day17_2_p300_R2.fastq.gz, Epithelial_BLM_Day17_2_H3_R1.fastq.gz, Epithelial_BLM_Day17_2_H3_R2.fastq.gz, Epithelial_BLM_Day17_2_H3K27ac_R1.fastq.gz, Epithelial_BLM_Day17_2_H3K27ac_R2.fastq.gz, Epithelial_BLM_Day17_2_p300_R1.fastq.gz, Epithelial_BLM_Day17_2_p300_R2.fastq.gz, Epithelial_CBP30_Day17_2_H3_R1.fastq.gz, Epithelial_CBP30_Day17_2_H3_R2.fastq.gz, Epithelial_CBP30_Day17_2_H3K27ac_R1.fastq.gz, Epithelial_CBP30_Day17_2_H3K27ac_R2.fastq.gz, Epithelial_GNE781_Day17_2_H3_R1.fastq.gz, Epithelial_GNE781_Day17_2_H3_R2.fastq.gz, Epithelial_GNE781_Day17_2_H3K27ac_R1.fastq.gz, Epithelial_GNE781_Day17_2_H3K27ac_R2.fastq.gz

Genome browser session
(e.g. UCSC)

NA

Methodology

Replicates

All CUT&Tag experiments were performed in duplicate.

Sequencing depth

CUT&Tag libraries were sequenced on a NovaSeq 6000 (Illumina) using the NovaSeq 6000 SP Reagent Kit v1.5 (100 Cycles) in paired-end mode (PE61, 61 bp for Read1 and Read2). The sequencing depth ranged from approximately 5M to 45M paired-end reads per sample, with an estimated library size between 1.9M and 32.7M unique reads.

Antibodies

Anti-H3K4me3 antibody (MAB Institute, MABI0304), Anti-H3K27me3 antibody (MAB Institute, MABI0323), Anti-H3 antibody (MAB Institute, MABI0301), p300 (Cell Signaling technology, #57625), and Anti-mouse IgG antibody (MAB Institute, ab46540).

Peak calling parameters

MACS2 (ver. 2.2.7.1) was used for peak calling with the following parameters: "-f BAM -g hs --nomodel", using the H3 CUT&Tag dataset as a control for each sample. For p300, peaks were called without a control sample under the same parameters.

Data quality

The sequencing reads were assessed for quality using fastp (v0.23.2) to remove adapter sequences and trim low-quality bases.

Software

Workflow management: Snakemake (v7.0.1), Snakemake wrapper (v2.1.0)
Sequence quality control & preprocessing: fastp (v0.23.2)
Read alignment: bowtie2 (v2.4.5)
Peak calling: MACS2 (ver. 2.2.7.1)
Read filtering & coverage estimation: samtools (v1.15.1), deeptools (v3.5.1)
Differential peak analysis: DiffBind (ver. 3.8.4)

Flow Cytometry

Plots

Confirm that:

☒ The axis labels state the marker and fluorochrome used (e.g. CD4-FITC).

☒ The axis scales are clearly visible. Include numbers along axes only for bottom left plot of group (a 'group' is an analysis of identical markers).

☒ All plots are contour plots with outliers or pseudocolor plots.

☒ A numerical value for number of cells or percentage (with statistics) is provided.

Methodology

Sample preparation	Cell suspensions were prepared using a flow cytometry buffer consisting of PBS with 1% BSA and 10 µM Y27632. The cells were incubated in Accutase (Innovative Cell Technologies, ICT-AT104-500-500) at 37 °C for 20 min and gently detached by pipetting. For staining, the cells were incubated with APC-conjugated anti-human CD54 antibody (BioLegend, 353112) for 20 min at 4 °C. Isolation of the Hoechsthigh cells was performed as previously described (Masui, A. et al. Stem Cell Reports. 2024.). Briefly, cells cultured on micro-patterned plates were first stained with Hoechst-33342 for 30 min at 37 °C, followed by incubation in Accutase at 37 °C for 20 min and dissociation by gentle pipetting. Hoechst-high populations were separated based on Hoechst fluorescence intensity using FACS.
Instrument	FACS Symphony S6 (BD Biosciences).
Software	FlowJo v10.8.1.
Cell population abundance	purity of single cell samples was manually determined.
Gating strategy	All analyses began with FSC vs. SSC gating to remove debris. Doublets were first excluded using FSC-W/FSC-H or FSC-H/FSC-A gating, followed by a second gating step using SSC-W/SSC-H or SSC-H/SSC-A to further eliminate doublets. Dead cells were excluded using PI or Sytox Blue staining, and GFP positivity was assessed using BB515. CD54$^+$ iATCs were gated and sorted using CD54-APC, while Hoechst-high iAT2s, iAT1s, and iATCs were gated and sorted using DAPI.

☒ Tick this box to confirm that a figure exemplifying the gating strategy is provided in the Supplementary Information.
